# Investigation of the Role of miR-1236-3p in Heat Tolerance of American Shad (*Alosa sapidissima*) by Targeted Regulation of *hsp*90*b*1

**DOI:** 10.3390/ijms26209908

**Published:** 2025-10-11

**Authors:** Mingkun Luo, Ying Liu, Wenbin Zhu, Bingbing Feng, Wei Xu, Zaijie Dong

**Affiliations:** 1 Key Laboratory of Freshwater Fisheries and Germplasm Resources Utilization, Freshwater Fisheries Research Center of Chinese Academy of Fishery Sciences, Ministry of Agriculture and Rural Affairs, Wuxi 214081, China; luomingkun@ffrc.cn (M.L.); zhuwb@ffrc.cn (W.Z.); 2 Wuxi Fisheries College, Nanjing Agricultural University, Wuxi 214128, China; lyly010307@163.com; 3 Fisheries Technology Extension Center of Jiangsu Province, Nanjing 210036, China; fbb-1981@163.com (B.F.); 13775663864@163.com (W.X.)

**Keywords:** *Alosa sapidissima*, miR-1236-3p, *hsp*90*b*1, heat stress

## Abstract

High temperatures are one of the most important abiotic stressors affecting the survival and growth of American shad (*Alosa sapidissima*). Building on previous omics sequencing studies of *A. sapidissima* liver and gills under high temperature stress, this study focused on investigating the regulatory role of miR-1236-3p and its target gene *hsp*90*b*1. The results indicate that the full-length cDNA of the *hsp*90*b*1 gene is 2023 bp and comprises a 5’ end of 58 bp, a 3’ end of 84 bp, and a coding region of 1881 bp, encoding 626 amino acids. Sequence alignment and phylogenetic tree analysis reveal that the *hsp*90*b*1 sequence is highly conserved across species. In situ hybridization showed that *hsp*90*b*1 is mainly localized in the cytoplasm. Software prediction identified a potential binding site between miR-1236-3p and *hsp*90*b*1. Through the construction of wild-type and mutant 3’UTR *hsp*90*b*1 dual luciferase reporter plasmids, the targeted relationship between the two was confirmed. In addition, the spatiotemporal expression levels of the *hsp*90*b*1 was found to be highest in the multicellular stage and liver tissue at a cultivation temperature of 27 °C; miR-1236-3P was highly expressed in the hatching stage and heart tissue at 30 °C. These findings provide a theoretical foundation for further investigating the regulatory role of non-coding RNA in *A. sapidissima* heat stress and offer data for subsequent molecular breeding studies.

## 1. Introduction

Fish are ectothermic animals, and temperature fluctuations not only affect their physiological functions, but also influence the concentration of dissolved oxygen, resulting in accelerated metabolism, increased oxygen consumption and antioxidant enzyme activity, which ultimately leads to oxidative stress [1]. As fish face global warming and environmental degradation, heat tolerance has gradually evolved into a crucial physiological trait affecting their survival and distribution [2,3]. When the ambient temperatures exceed the optimal range for fish, the fish show a thermal stress reaction that affects their growth, metabolism, development, survival and reproduction [4,5]. In a high-temperature experiment with juvenile flounders (*Paralichthys olivaceus*), for example, heat shock treatment led to a significant increase in the expression of several immune-related genes such as interleukin-8 (*IL*-8), c-lysozyme (*c-Lys*) and immunoglobulin M (*IgM*)) in the liver and brain, which activated the immune system of the fish and increased their tolerance to high temperatures [6]. Low temperatures reduce the fluidity of tilapia cell membranes and enzyme activity, leading to slow growth, lethargy and even death. In response to cold stress, fish increase their unsaturated fatty acid content to improve membrane fluidity and alter enzyme activity in the body to maintain physiological functions [7]. Fang et al. [8] found that the gut microbiota composition and metabolic profile of tsinling lenok trout (*Brachymystax lenok tsinlingensis* Li) changed significantly under heat stress conditions, with a marked decrease in the amount of glutathione, which is synthesized from glutamic acid and glycine, affecting growth and immune function. Heat shock proteins are protective proteins synthesized by cells in response to environmental factors such as temperature, salinity or dissolved oxygen content, and divided into six families based on their molecular weight: HSP40, HSP60, HSP70, HSP90, HSP110 and low molecular weight HSPs, and are often used as effective biomarkers of environmental stress and enable the assessment of the severity of damage in fish [9]. HSP70 is a highly efficient molecular chaperone that protects cells from damage caused by stress stimuli. Padmini et al. [10] found, all markers for oxidative and nitrosative stress as well as the expression of HSP70 in *Mugil cephalus* showed significant seasonal variations, peaking in summer, and indicates that HSP70, which is overexpressed in fish hepatocytes under stress, can promote cell survival by counteracting the changes caused by oxidative and nitrosative stress. Studies on striped pufferfish (*Takifugu fasciatus*) revealed that HSPs exhibit different expression patterns under different stress conditions following exposure to low temperatures and biological stressors, and HSPs showed a sustained response to both cold stress and infection with *Aeromonas hydrophila* [11]. In our previous study on American shad (*Alosa sapidissima*) [12], we also observed a significant increase in *hsp*90*b*1 expression with increasing temperature, suggesting its potential protective role in thermal stress responses of fish. However, the mechanisms underlying the involvement of *hsp*90*b*1 in the regulation of thermal stress and its interactions with other genes require further investigation.

*A. sapidissima* belongs to the order Clupeiformes, family Clupeidae, genus Alosa, and its morphology, body shape and nutritional value are highly similar to those of the Chinese shad (*Tenualosa reevesii*), which led to its introduction and gradual bred in China at the end of the 20th century [13,14]. *A. sapidissima* is a warm-water fish species with an optimal temperature range of 20~26 °C, and its growth and reproduction are significantly affected by fluctuations in water temperature [15]. Our previous studies found that the optimal incubation temperature for *A. sapidissima* is 18~20 °C. The blastula stage is a critical phase in the embryonic development of allis shad. From this stage onwards, gene expression related to cell formation and cell division is downregulated, while gene expression related to organogenesis, cell differentiation and other functional processes is significantly upregulated [16]. Furthermore, we performed multi-omics analysis on liver and gill tissue of *A. sapidissima* at different rearing temperatures and identified a large number of miRNAs (e.g., miR-125b, miR-92b), mRNAs (e.g., *cat*, *alpl*, etc.), proteins (e.g., HSP70, HSP90B1 and HSP5, etc.) and metabolites (e.g., L-serine, L-isoleucine, L-cystine, choline and betaine, etc.), suggesting that they can adapt to environmental changes under high temperature stress conditions by regulating gene expression and metabolic pathways [12,17,18]. This also lays the foundation for further investigation into the molecular regulatory mechanisms of these miRNAs and their target genes.

MicroRNAs (miRNAs) are a class of important endogenous, non-coding small RNAs, typically 18 to 30 nucleotides in length, that are widely distributed in eukaryotic cells [19]. miRNAs regulate gene expression through complementary pairing with target mRNAs and are involved in diverse biological processes, including cell proliferation, differentiation, metabolism and stress responses [20,21]. During early development of zebrafish (*Danio rerio*), miR-181a-5p, which regulates mitochondrial biogenesis and respiration, was significantly upregulated in juvenile fish exposed to temperature fluctuations (28 ± 5 °C), suggesting that early-life exposure to diel thermal fluctuations induces lasting epigenetic changes [22]. In addition, Liu et al. [23] found that the relative expression of miR-301a-3p in rainbow trout (*Oncorhynchus mykiss*) was significantly lower at 24 °C than at 18 °C, while the target gene *hsp*90*b*2 showed the opposite pattern. The aim of this study is to further validate the target genes of miR-1236-3p based on previous studies and to evaluate the potential role of miR-1236-3p in the liver of *A. sapidissima* by inhibiting *hsp*90*b*1 under heat stress conditions. The results of this study will provide useful information on the response of miRNAs in the liver of *A. sapidissima* under heat stress conditions and serve as a reference for future heat stress research in this species.

## 2. Results

### 2.1. Cloning and Sequence Characterization of the hsp90b1 Gene

Using RACE amplification, we successfully cloned the 2023 bp cDNA of the full-length *hsp*90*b*1 gene from the *A. sapidissima*, which comprises a 58 bp 5’ untranslated region (5’-UTR), an 84 bp 3’ untranslated region (3’-UTR), and a 1881 bp coding sequence (CDS). The CDS starts with the ATG codon and ends with the TGA codon and encodes a total of 626 amino acids (Figure S1). The prediction of the ProtParam software (https://web.expasy.org/protparam/) shows that the amino acid sequence of the HSP90B1 protein has a relative molecular mass of 71,975.34 Da, with a theoretical isoelectric point (pI) of 4.96. This sequence consists predominantly of hydrophilic amino acids containing 124 negatively charged residues (Asp + Glu) and 93 positively charged residues (Arg + Lys), which classifies it as an unstable hydrophilic protein (Figure S2a). The prediction of SignalP 4.0 software suggests that the first 21 amino acids of the protein sequence could form a signal peptide (Figure S2b); and no significant transmembrane structures are present (Figure S2c). Prediction of the secondary structure of the HSP90B1 protein revealed 50.80% (318) as *α*-helices, 14.86% (93) as extended strands, 3.99% (25) as *β*-turns, and 30.35% (190) by random coils (Figure S3).

**Figure 1:**
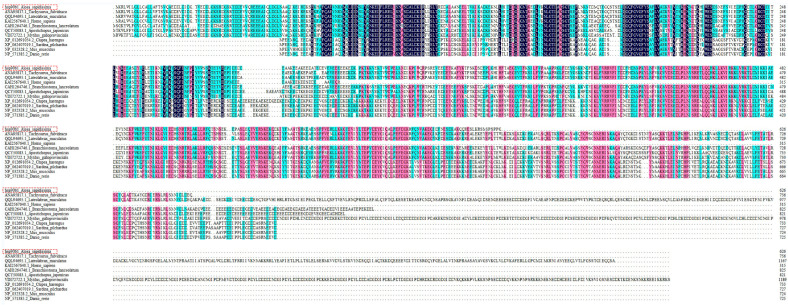
Amino acid sequence alignment for *A. sapidissima hsp*90*b*1 and other species. Different colors indicate the similarity of the amino acid sequences: black: 100%; pink: 70%; cyan: 50% and more; white: below 50%. The red dashed box indicates the *hsp*90*b*1 gene information for the *A. sapidissima*.

By comparing the amino acid sequence encoded by the *hsp*90*b*1 gene of the *A. sapidissima* with those of 10 other species, we found that the *A. sapidissima* has the highest homology with the *Tachysurus fulvidraco* at 89.71%, and a similarity of 88.10% and 85.61% with those of the *Lateolabrax maculatus* and *Homo sapiens*, respectively, and the similarity with those of the *Clupea harengus*, *Sardina pilchardus*, *Mus musculus* and *Danio rerio* was between 45% and 50% (Figure 1).

**Figure 2:**
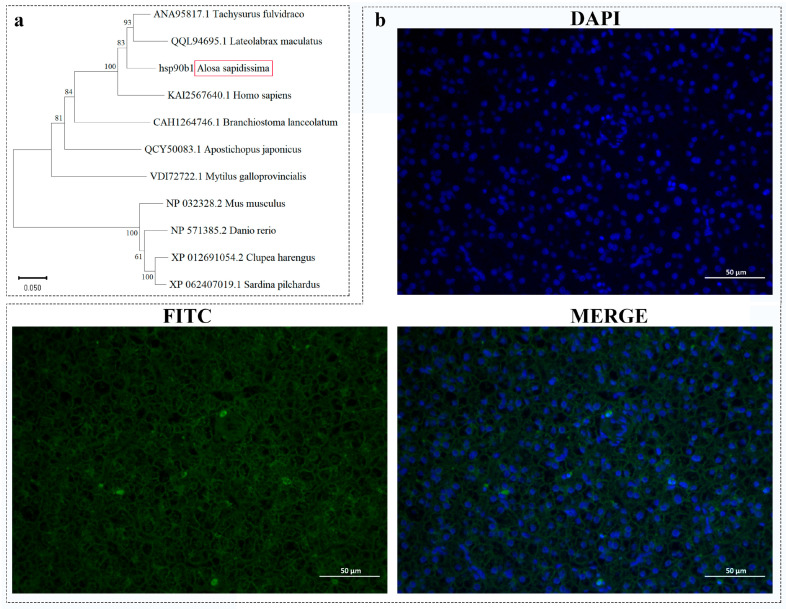
Analysis of the sequence features of the *hsp*90*b*1 gene in the *A. sapidissima*. (**a**) Phylogenetic tree of HSP90B1 amino acid sequence of *A. sapidissima*. (**b**) Results of in situ hybridization analysis of the *hsp*90*b*1 gene in *A. sapidissima* (×400, 50 μm), with nuclei stained with DAPI (blue) and *hsp*90*b1* signals stained with FITC (green). The red box represents the *A. sapidissima*.

The results of the phylogenetic tree generated by the NJ method (Figure 2a) show that *A. sapidissima* forms a cluster with the *Tachysurus fulvidraco* and the *Lateolabrax maculatus*, indicating a close phylogenetic relationship. The function of the *hsp*90*b*1 gene is closely linked to its subcellular localization. To further clarify the role in the liver, we investigated its cellular distribution by fluorescence in situ hybridization, and the results showed that the *hsp*90*b*1 gene is mainly distributed in the cytoplasm of liver tissue cells (Figure 2b).

### 2.2. Association Analysis Between miR-1236-3p and hsp90b1

**Figure 3:**
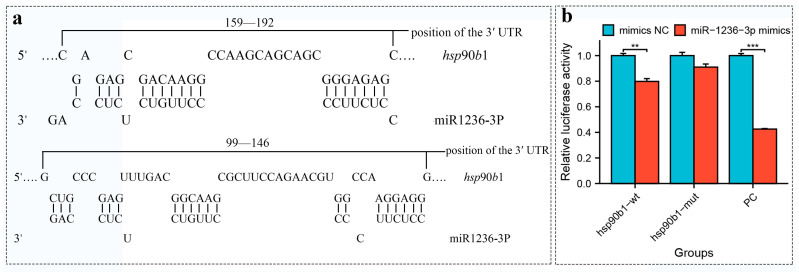
(**a**) Binding site analysis between *hsp*90*b1* gene and miR-1236-3p. (**b**) Analysis of the target relationship of miR-1236-3p with *hsp*90*b1* in HEK 293T cells using a dual luciferase reporter assay. Relative luciferase reporter expression was normalized to NC. Each treatment was repeated in triplicate. Data are presented as mean ± standard error. An asterisk (**) indicates *p* < 0.01 compared to the NC mimic, and “***” indicates *p* < 0.001.

Through target gene software prediction analysis, we identified two complementary sites in the 3’ UTR region of the *hsp*90*b*1 gene (...5’...GAG.GACAAGG...GGGAGAG...3’, ...5’...GGCAAG...GG.AGGAGG...3’...) that specifically bind miR-1236-3p (Figure 3a). We then performed a dual luciferase reporter assay to further test the relationship between them. By measuring the cell density of the group expressing the fluorescently labeled gene (PE-GFP), we indirectly evaluated the transfection efficiency. The results showed that the transfection efficiency was above 80% in all groups, indicating successful transfection of the plasmid (Figure S4). The results of luciferase activity detection are shown in Figure 3b. miR-146b decreased the fluorescence activity of the *traf*6 3’ UTR group by approximately 57% compared to miR-146b-NC, indicating that the experimental system was functioning normally. Recombinant plasmids *hsp*90*b*1-wt and *hsp*90*b*1-mut were also co-transfected into HEK 293T cells with miR-1236-3p mimic or mimic NC. Luciferase expression in the mimic NC + *hsp*90*b*1-wt and mimic NC + *hsp*90*b*1-mut groups showed no significant changes (*p* > 0.05). Co-transfection of miR-1236-3p-mimic with *hsp*90*b*1-wt resulted in inhibition of luciferase activity (*p* < 0.05), but co-transfection of miR-1236-3p mimic with *hsp*90*b*1-mut did not significantly affect luciferase activity (*p* > 0.05). These results suggest that miR-1236-3p is involved in the regulation of *hsp*90*b*1 expression by binding to the putative binding site in the 3’-UTR of the *hsp*90*b*1 gene.

### 2.3. Spatiotemporal Expression Properties of the hsp90b1 and miR-1236-3p

**Figure 4:**
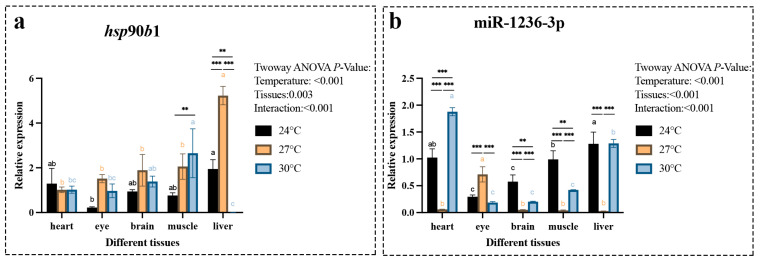
Expression level of the *hsp*90*b*1 gene (**a**) and miR-1236-3p (**b**) in different tissues of the *A*. *sapidissima* under various temperature conditions. All parameter values represent the mean ± standard error of three replicate groups. Different letters within the same color column indicate significant differences between tissues (*p* < 0.05); “**” indicates significant differences (*p* < 0.01) within the same group across different temperature conditions, while “***” indicates p < 0.001.

To further investigate the expression patterns of the *hsp*90*b*1 gene and miR-1236-3p, we systematically analyzed their expression levels in different tissues, temperature conditions and early developmental stages. The results showed significant differences in the overall expression trends of *hsp*90*b*1 gene and miR-1236-3p in various tissues under different temperature conditions. In particular, the expression of *hsp*90*b*1 gene was highest in liver tissue at 27 °C, followed by significantly reduced expression at 30 °C (Figure 4a). Comparison of *hsp*90*b*1 expression in different tissues at the same temperature revealed significantly higher levels in muscle tissue at 30°C compared to other tissues, while liver tissue had significantly higher expression than other tissues at 27 °C (Figure 4a). In addition, the expression of miR-1236-3p was significantly higher in heart tissue at 30 °C compared to other tissues and in liver tissue at 24 °C compared to other tissues (Figure 4b). At 27 °C, the expression of miR-1236-3p increased significantly in the eye, while it decreased extremely in the liver tissue (Figure 4b).

**Figure 5:**
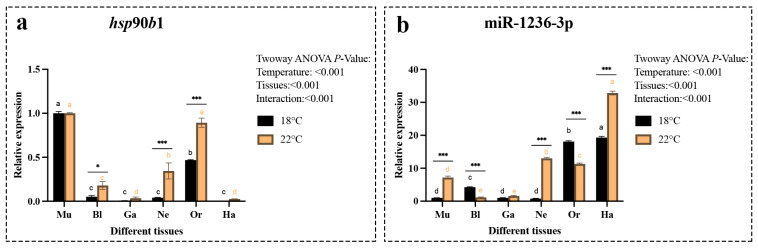
Expression level of the *hsp*90*b*1 gene (**a**) and miR-1236-3p (**b**) during different early developmental stages at 18 °C and 22 °C. All parameter values represent the mean ± standard error of three replicate groups. Different letters within the same color column indicate significant differences between developmental stages (*p* < 0.05); “*” denotes significant differences between temperature treatments at the same developmental stage (“*” means *p* < 0.05, “***” means *p* < 0.001). Multicellular stage (Mu), blastula stage (Bl), gastrula stage (Ga), neurula stage (Ne), organogenesis stage (Or) and hatching stage (Ha).

We then examined the expression levels of *hsp*90*b*1 and miR-1236-3p during the major developmental stages of *A*. sapidissima at two different temperatures: 18 °C and 22 °C. The results showed that the gene expression of *hsp*90*b*1 peaked during the multicellular embryonic stage, followed by a significant decrease. It then increased significantly during the organogenesis stage before decreasing significantly again (Figure 5a). The expression level of miR-1236-3p remained low during the multicellular, blastula and gastrula stages. At 22 °C, it started to increase significantly during the neural crest stage. Subsequently, it gradually increased at both 18 °C and 22 °C during the organogenesis stage and reached its highest expression level during the hatching stage (Figure 5b).

## 3. Discussion

Temperature is a crucial abiotic factor for aquatic organisms, affecting their embryonic development, physiological functions, survival and ability to maintain homeostasis in response to external stressors [24]. In view of the worsening effects of global climate change, the regulatory mechanisms of heat tolerance in aquatic organisms such as fish have become an important field of research [25]. Heat stress induces the production of heat shock proteins, which improve the ability of body cells to respond to external environmental stress, especially heat tolerance [26]. The *hsp*90*b*1 gene, a potential candidate and heat stress marker, maintains normal endoplasmic reticulum function by activating the response mechanism to unfolded proteins [27]. Thus, the expression of *hsp*90*b*1 was significantly increased under heat stress conditions in mice [28], broiler chickens [29] and rainbow trout [30]. Our previous studies [12,17] have also shown that the expression of *hsp*90*b*1 is closely correlated with temperature changes and increases significantly under high temperatures, suggesting that *hsp*90*b*1 may play an important protective role in the response of fish heat stress.

In this study, we cloned the complete cDNA sequence of the *A. sapidissima hsp*90*b*1 gene, which encodes 626 amino acids. We found that the protein encoded by this gene is a non-transmembrane protein that may be involved in biological processes such as transport and metabolism [31]. The secondary structure prediction suggests that the HSP90B1 protein consists of more than 50% *α*-helical domains. *α*-helices fulfill several functions in proteins: they maintain protein stability through intramolecular hydrogen bonding, ensure that proteins maintain their correct conformation under physiological conditions, and participate in protein-protein and protein-DNA interactions [32,33]. Furthermore, sequence alignments and phylogenetic tree between different species showed that the amino acid sequence of *A. sapidissima hsp*90*b*1 is highly conserved. Among them, we found that it is distantly related to zebrafish, which may be related to cumulative variations at key gene loci [34]. The results of in situ hybridization show that the *hsp*90*b*1 gene is mainly localized in the cytoplasm of liver tissue. This finding is consistent with the studies of Audouard et al. [35], who reported high cytoplasmic expression of *hsp*90*b*1 in mouse cells. However, the specific molecular mechanism of its action requires further research.

Subsequently, we confirmed that miR-1236-3p can target and regulate *hsp*90*b*1 gene through bioinformatic software predictions and dual luciferase reporter assays, suggesting a potential interaction between the two genes that jointly influences the response of *A. sapidissima* to high-temperature stress. According to relevant studies, *hsp*90*b*1 is not only highly expressed in response to thermal stress, but is also closely associated with several intracellular signaling pathways that are critical for tumorigenesis and progression [36,37]. In nasopharyngeal carcinoma, for example, the expression of *hsp*90*b*1 is closely associated with tumor proliferation, glycolysis and angiogenesis. Inhibition of *hsp*90*b*1 leads to a significant reduction in tumor cell proliferation and the ability to metastasize [38]. Studies suggest that *hsp*90*b*1 is highly expressed in various cancers, with its overexpression often associated with poor prognosis [39]. Furthermore, *hsp*90*b*1 is involved as a molecular chaperone in processes such as protein refolding, environmental adaptation and immune responses and plays a fundamental role in the defense against various biotic and abiotic stress factors and in the maintenance of cellular homeostasis [40]. At the same time, *hsp*90*b*1 plays a crucial role in the cellular antioxidant stress response by helping cells resist oxidative damage and maintaining the stability of the intracellular environment by regulating the redox balance [41]. Saleh et al. [42] demonstrated that silencing the *hsp*90 gene in *Trichodina* parasites significantly inhibits their growth and development. In experiments with common carp (*Cyprinus carpio*), spore-forming parasites treated with antisense oligonucleotides showed significantly reduced infectivity compared to the control group. This provides genetic evidence for the critical role of *hsp*90 in parasite growth and development and suggests that *hsp*90 could serve as a novel therapeutic target for successful disease control. In osteosarcoma research, qPCR analysis of miR-1236-3p expression revealed significant lower levels in osteosarcoma tissue compared to normal cartilage tissue, suggesting that miR-1236-3p may exert tumor suppressive effects [43]. These results indicate that *hsp*90*b*1 and miR-1236-3p play a critical role in cancer development and response to environmental stress. In addition, this study shows that both also exert key functions in heat stress responses; however, the specific molecular mechanisms and regulatory signaling pathways require further investigation.

In oviparous vertebrates, two critical events may occur between successful fertilization and hatching: sperm penetration of the egg, such as zona pellucida dissolution [44]; and the blastocyst stage during hatching, where embryo-derived cathepsins accelerate animal hatching [45]. In this study, we found that *hsp*90*b*1 exhibited the highest expression levels during the multicellular stage of embryonic development and in the liver at 27 °C. Since the multicellular stage is a critical period for neural crest differentiation, this suggests that the *hsp*90*b*1 gene may be involved in the process of neural crest differentiation [46]. In addition, *hsp*70 and *hsp*90 can regulate the expression of cellular heat shock proteins and are thought to contribute to abnormal embryonic development during incubation [47]. In this study, their expression levels were found to increase significantly when embryonic development progressed to the organogenesis stage, suggesting that HSP90B1 may be involved in the developmental processes of embryonic tissues and organs. miR-1236-3p has the highest expression levels during the hatching stage and in the heart at 30 °C. Its expression patterns during the developmental stages suggest that it plays a crucial role in the development and growth of *A. sapidissima* after hatching. Moreover, its expression level remains relatively high during the organ formation stage, suggesting that miR-1236-3p, like the *hsp*90*b*1 gene, may be involved in early embryonic development and play an important role in the formation of embryonic tissues and organs. These findings provide a theoretical basis for further research into the regulatory role of the hsp90b1 gene and miR-1236-3P during thermal stress in *A. sapidissima*.

## 4. Materials and Methods

### 4.1. Ethical Statement

This study was approved by the Bioethical Committee of the Freshwater Fisheries Research Center (FFRC) of the Chinese Academy of Fishery Sciences (CAFS) (BC2013863, 9/2013), and the fish were handled in accordance with the relevant guidelines of the Ministry of Science and Technology, Beijing, China (No. 398, 2006).

### 4.2. Experimental Fish and Sample Collection

The general experimental design of this study builds on our previous research on *A. sapidissima* [12]. The experimental fish were artificially bred second-year *A. sapidissima* obtained from the Yangzhong Base of Jiangsu Provincial Fisheries Technology Promotion Center. The water temperature was monitored throughout the experiment using a temperature control system. The different temperature gradients included a low temperature group (24.0 ± 0.5 °C), a medium temperature group (27.0 ± 0.5 °C) and a high temperature group (30.0 ± 0.5 °C). Shad of similar body size, without external injuries and with a robust body structure (average weight 354 ± 35 g) were selected. During sampling, three individuals were randomly selected from each temperature group. After anesthesia with phenoxyethanol (0.3 mL/L), liver, brain, heart, muscle and eye tissues were collected, washed with physiological saline, rapidly frozen in liquid nitrogen and brought to −80 °C for storage. In addition, two *A. sapidissima* were selected from the low-temperature group: one liver tissue sample was frozen in liquid nitrogen for gene cloning, and the other liver tissue sample was placed in 4% paraformaldehyde for in situ hybridization analysis. In addition, according to our previous embryonic stage sampling method [48], samples were collected at the multicell (Mu), blastula (Bl), gastrula (Ga), neurula (Ne), organogenesis (Or) and hatching (Ha) stage under incubation conditions of 18 °C and 22 °C. These samples were quickly frozen in liquid nitrogen and brought to −80 °C for storage.

### 4.3. Cloning and In Situ Hybridization (ISH) Analysis

Based on the information about the *hsp*90*b*1 gene from previous transcriptomic data [12], the open reading frame (ORF), 5′ and 3′ regions were amplified using the RACE method referring to [49]. Specific primers were designed using Primer Premier 5 (Table S1), and the *A. sapidissima hsp*90*b*1 gene sequence was amplified using a SMARTer™ RACE cDNA Amplification Kit (Clontech, Palo Alto, CA, USA). ProtScale (http://web.expasy.org/protscale/, 1 July 2024) was used to analyze the hydrophilicity/hydrophobicity of amino acid residues, ProtParam (https://web.expasy.org/protparam/, 10 July 2024) to predict the theoretical isoelectric point of protein molecules, DNAMAN 8.0 (Lynnon Co., Pointe-Claire, Canada) for alignment and multiple comparison of sequences and MEGA 7.0 [50] to construct phylogenetic trees. The *ISH* probe was designed based on the cDNA sequences of the *hsp*90*b*1 gene with the sequences 5′-FAMCUUCUCCUUGUCAGCCUUAAUCUUCACGGUCAGU-3′. ISH analysis in different tissues was performed according to the previously method of Thisse et al. [51].

### 4.4. Dual-Luciferase Reporter Assays

In our previous study [12], we found by RNA-sequencing that the expression levels of miR-1236-3p and *hsp*90*b*1 in *A. sapidissima* liver differ at various temperatures. Here, we used TargetScan (http://www.targetscan.org, 5 July 2024) and RNAhybrid (https://omictools.com/rnahybrid-tool, 5 July 2024) to predict the binding site information. The 3’UTR region of the *hsp*90*b*1 gene was synthesized and cloned into the pmirGLO vector (Promega, Madison, WI, USA), downstream of the luciferase minigene. We then mutated the putative miRNA target sequence within the 3’UTR using the Site-Directed Mutagenesis Kit (Umibio, Shanghai, China).

Detailed information about the dual luciferase reporter assay can be found in our previous study [52]. More specifically, as follows: HEK 293T cells were seeded at a density of 1.0 × 10^5^ cells per well in a 48-well culture plate and cultured for 24 hours before transfection. The Lipofectamine 2000 reagent kit (Invitrogen, Carlsbad, CA, USA) was then used to co-transfect pmirGLO-*hsp*90*b*1-wt or pmirGLO-*hsp*90*b*1-mut plasmids with miR-1236-3p mimics or mimic negative controls (NC) into HEK293T cells according to the manufacturer’s instructions. In addition, miR-146b and *traf*6 genes were used as positive controls (PC). 48 hours after transfection, luciferase assays were performed using the Dual-Glo^®^ Luciferase Assay System (Promega, WI, USA) according to the manufacturer’s instructions. Data collection methods were previously described in [53]. *Renilla* luciferase activity was normalized to *firefly* luciferase activity and expressed as a percentage of the control. Three biological replicates were used for each treatment.

### 4.5. RNA Isolation and Quantitative PCR

MiRNAs were extracted using a miRNeasy kit (Takara, Kyoto, Japan) and RNA was extracted using TRIzol^®^ reagent (CWBio, Suzhou, China) according to the manufacturer’s protocol. The concentration was measured using a NanoDrop 2000UV-spectrophotometer (Thermo, Waltham, MA, USA), and the quality and integrity were checked by OD 260/280 and 1% agarose gel electrophoresis. Total RNA was reverse transcribed using Prime-Script RT Master Mix (Takara, Kyoto, Japan) and miRNAs were reverse transcribed using Mir-XTM miRNA First-strand Synthesis Kit (Takara, Kyoto, Japan). Primers were designed using Primer Premier 5 (Table S1). qRT-PCR was performed using SYBR Premix Ex TaqII (Takara, Kyoto, Japan) on a CFX-96 Real-time PCR System (Bio-Rad, Berkeley, CA, USA). All samples were run in triplicate, and the relative level of miRNAs and mRNAs was normalized to the amount of *U6* and *β-actin*, respectively. Relative expression was calculated using the comparative threshold cycle (C_T_) method [54], referred to as the 2^−ΔΔCT^ algorithm.

### 4.6. Statistical Analysis

All results were expressed as means ± standard error of the mean. Statistical analysis was performed using SPSS 20.0 (SPSS Inc., Chicago, IL, USA). Prior to statistical analysis, all data were validated for normality (Shapiro–Wilk test) and homogeneity of variance (Levene test). The independent and interactive effects of dietary energy content and feeding regime were analyzed using a two-way ANOVA.

## 5. Conclusions

In this study, we successfully cloned the full-length *hsp*90*b*1 gene from *A. sapidissima* for the first time and found that it is mainly localized in the cytoplasm. Under heat stress conditions, the expression of *hsp*90*b*1 increases, while the expression of miR-1236-3p shows the opposite trend. Possibly, there is a negative feedback relationship between miR-1236-3p and *hsp*90*b*1, which is achieved by binding to the 3′-UTR regions of *hsp*90*b*1. Our results provide evidence for the role of miR-1236-3p in the liver and contribute to the understanding of miRNA-mediated gene regulatory mechanisms in American shad under heat stress.

## Data Availability

The original contributions presented in this study are included in the article/Supplementary Material. Further inquiries can be directed to the corresponding author.

## Supplementary Materials

The following supporting information can be downloaded at https://www.mdpi.com/article/10.3390/ijms26209908/s1.

## Abbreviations

The following abbreviations are used in this manuscript:

HSP	Heat shock protein
miRNA	MicroRNAs
CDS	Coding sequence
UTR	Untranslated region
NJ	Neighbor joining
qRT-PCR	Real-time quantitative reverse transcription polymerase chain reaction
