# Supplementary material for: Investigation of the Role of miR-1236-3p in Heat Tolerance of American Shad (Alosa sapidissima) by Targeted Regulation of hsp90b1
Source: Int J Mol Sci. 2025 Oct 11;26(20):9908. doi: 10.3390/ijms26209908 (PMC12564195; doi:10.3390/ijms26209908)
Supplement: Supplementary file 1 [file ijms-26-09908-s001.zip › Figure.s1.pdf]

1 gcagatcccatccgaaggactgtaaaaggcatcctgaagtgaattttacttcatcaaaATGAAGCGTTTATGGATCTTAGGTCTTCTATGT
1 M K R L W I L G L L C
92 GCTCTTCTTGCCTTCACATCTGTGCAGGCAGAAGATGATCTTGACATTGACGGAACCGTGGAGGACGATCTAGGAAAAAGCAGAGATGGT
12 A L L A F T S V Q A E D D L D I D G T V E D D L G K S R D G
182 TCCAAGACAGATGATGAAGTTGTACAAAGGGAGGAAGAAGCTATTCAACTGGATGGATTGAACGCAGCGCAGATTAAGGAAATTCGAGAT
42 S K T D D E V V Q R E E E A I Q L D G L N A A Q I K E I R D
272 AAGTCGGAAAAGCATGCCTTCCAGGCTGAGGTCAACCGCATGATGAAGCTCATCATCAACTCCCTGTACAAGAACAAGGAGATCTTCTTG
72 K S E K H A F Q A E V N R M M K L I I N S L Y K N K E I F L
362 AGGGAGCTCATCTCCAACGCTTCTGACGCCCTGGACAAGATCCGCTTGCTCTCGCTGACCAGGGATGATGCCTTGGCTGCCAACGAAGAA
102 R E L I S N A S D A L D K I R L L S L T R D D A L A A N E E
452 CTGACCGTGAAGATTAAGGCTGACAAGGAGAAGAACCTGCTCCACATCACCGACACAGGCATCGGCATGACCAAAGAGGATCTGGTGAAG
132 L T V K I K A D K E K N L L H I T D T G I G M T K E D L V K
542 AACCTGGGAACCATCGCCAAGTCCGGCACCAGCGAATTCCTCAACAAGATGACCGACATGCAGACAGAGGGCCAGTCCACGTCCGAGCTG
162 N L G T I A K S G T S E F L N K M T D M Q T E G Q S T S E L
632 ATTGGCCAGTTCGGCGTTGGCTTCTACTCCGCCTTCCTGGTGGCCGACAAGGTCATCGTCACGTCCAAGCACAACAACGGCACGCAGCAC
192 I G Q F G V G F Y S A F L V A D K V I V T S K H N N G T Q H
722 ATCTGGGAGTCGGACTCCAACGAGTTTTCAGTCATCGAGGACCCGCGCGGAGACACCCTGGGGCGCGGAACTACCATCACGCTCGTGATG
222 I W E S D S N E F S V I E D P R G D T L G R G T T I T L V M
812 AAAGAGGAGGCCTCTGATTACCTGGAGCTGGAGACCATTAAGAACCTGGTCAGGAAGTACTCGCAGTTCATCAACTTCCCCATCTACGTC
252 K E E A S D Y L E L E T I K N L V R K Y S Q F I N F P I Y V
902 TGGAGCAGCAAGACGGAAACGGTAGAGGAGCCCATCGATGAGGATGACGAGGCTGCCAAGGAGGAGGCCAAAGAGGAGGACGCCACTGAG
282 W S S K T E T V E E P I D E D D E A A K E E A K E E D A T E
992 GATGAGGTAGAGGTGGAGGAGGAAGAGGAGAAGGAGGACAAGCCCAAGACCAAGAAGGTGGAGAAGACGGTGTGGGACTGGGAGCTGATG
312 D E V E V E E E E E K E D K P K T K K V E K T V W D W E L M
1082 AACGACATTAAGCCCATCTGGCAGCGCCCCTCTAGGGAGGTGGAGGAGGACGAGTATAAGGCCTTCTACAAGACCTTCTCCAAGGACTCG
342 N D I K P I W Q R P S R E V E E D E Y K A F Y K T F S K D S
1172 GACGAGCCCCTTGGTCACATCCACTTCACGGCGGAGGGAGAGGTCACCTTTAAGTCCATCCTGTTTGTGCCCGCTGCTGCTCCGCGTGGC
372 D E P L G H I H F T A E G E V T F K S I L F V P A A A P R G
1262 CTTTTTGATGAATACGGCTCCAAGAAGAATGACTTCATCAAGCTGTTTGTACGCAGAGTGTTCATCACTGATGATTTCCACGACATGATG
402 L F D E Y G S K K N D F I K L F V R R V F I T D D F H D M M
1352 CCCAAGTACCTCAACTTTGTCAGGGGTGTGGTGGACTCTGATGACCTGCCCCTCAACGTCTCCAGGGAGACCCTGCAGCAGCACAAACTG
432 P K Y L N F V R G V V D S D D L P L N V S R E T L Q Q H K L
1442 CTGAAGGTCATCCGCAAGAAGCTGGTGCGCAAGACCCTGGACATGATCAAGAAGATC GCCGAGGAGGTGTACAACGAGAAGTTCTGGAAG
462 L K V I R K K L V R K T L D M I K K I A E E V Y N E K F W K
1532 GAGTTTGGCACCAACATCAAGCTGGGCGTGATCGAGGACCACTCCAACCGCACCCGCCTGGCCAAGCTGCTGCGCTTCCAGACGTCCAAC
492 E F G T N I K L G V I E D H S N R T R L A K L L R F Q T S N
1622 AGCGAGAAGGAGCCGGCCAGCCTGGAGCAGTATGTGGAGAGGATGAAGGAGAAGCAGGACAAGATCTACTTCATGGCTGCCACCAGCAGG
522 S E K E P A S L E Q Y V E R M K E K Q D K I Y F M A A T S R
1712 AAGGAGGCCGAGTCTTCTCCCTTCGTTGAGCGGCTGCTGAAGAAGGGCTATGAGGTGATCTACCTGACGGAGCCCGTGGACGAGTACTGC
552 K E A E S S P F V E R L L K K G Y E V I Y L T E P V D E Y C
1802 GCCAAGCAGCAGCGGGAGAGCCTGAAAAGGAGTATGAGCCCCTCACCACCTGGATGAATTGACTTCAACGAGAGCGACAAGGCCAAGCAG
582 I Q A L P E F D G K R F Q N V A K E G I D F N E S D K A K Q
1892 CAGCGGGAGAGCCTGAAAAGGAGTATGAGCCCCTCACCACCTGGATGAaggacaacgccctcaaggacagatcgagaaggctgtgctgtc
612 Q R E S L K R S M S P S P P G *
1982 ccagagactgaccactccccctgcgcttctggtggccagtca
