# Supplementary material for: Investigation of the Role of miR-1236-3p in Heat Tolerance of American Shad (Alosa sapidissima) by Targeted Regulation of hsp90b1
Source: Int J Mol Sci. 2025 Oct 11;26(20):9908. doi: 10.3390/ijms26209908 (PMC12564195; doi:10.3390/ijms26209908)
Supplement: Supplementary file 1 [file ijms-26-09908-s001.zip › supplementary captions.pdf]

Supplementary Figure

Figure S1

The cDNA sequence of the *A. sapidissima hsp90b1* gene and the amino acid sequence it encodes. Upper case, lower case and italicized letters represent the coding sequence (cds), the non-coding sequence and the signal peptide sequence, respectively. The corresponding coded amino acids are indicated below the nucleotides, and * stands for the stop codon.

Figure S2

Amino acid sequence analysis of *hsp90b1*. (a) prediction of hydrophilicity; (b) prediction of signal peptide; (c) prediction of transmembrane structure.

Figure S3

Prediction of the secondary structure of the *A. sapidissima* HSP90B1 protein. Blue indicates the α-helix; red, the extended sheet; green, the *β*-turn; purple the irregular coil.

Figure S4

Representative images of plasmid transfection efficiency after 24 h of 293T cell culture. Left and right correspond to white light photo of NC control 293T cells and fluorescence photo of plasmid single transfer group with PE-GFP gene (100×), respectively.
