# Supplementary material for: Investigation of the Role of miR-1236-3p in Heat Tolerance of American Shad (Alosa sapidissima) by Targeted Regulation of hsp90b1
Source: Int J Mol Sci. 2025 Oct 11;26(20):9908. doi: 10.3390/ijms26209908 (PMC12564195; doi:10.3390/ijms26209908)
Supplement: Supplementary file 1 [file ijms-26-09908-s001.zip › Table s1.pdf]

Table S1. Primers used in this study

Primer name	Sequence 5' to 3'	Application
*hsp*90*b*1-F	GTGCTCTTCTTGCCTTCACATC	ORF sequence amplification
*hsp*90*b*1-R	AATCTGCGCTGCGTTCAATC	
5' inner	GCTACGTAACGGCATGACAGTG	5'-RACE
5' outer	GCTGTCAACGATACGCTACGTAAC	
3' inner	GAGGAGCCCATCGATGAGGATGACGAGG	3'-RACE
3' outer	TCGACTACAGATCGGCCAT	
miR-1236-3p	CCTCTTCCCCTTGTCTCTC	qRT-PCR
*hsp*90*b*1-F	GTGCTCTTCTTGCCTTCACATC	
*hsp*90*b*1-R	AATCTGCGCTGCGTTCAATC	
U6-F	GGAACGATACAGAGAAGATTAGC	
U6-R	TGGAACGCTTCACGAATTTGCG	
β-actin-F	GCCCCACCTGAGCGTAAATA	
β-actin-R	GAGTCGGCGTGAAGTGGTAA	
