# Supplementary figures and images for: Investigation of the Role of miR-1236-3p in Heat Tolerance of American Shad (Alosa sapidissima) by Targeted Regulation of hsp90b1
Source: Int J Mol Sci. 2025 Oct 11;26(20):9908. doi: 10.3390/ijms26209908 (PMC12564195; doi:10.3390/ijms26209908)

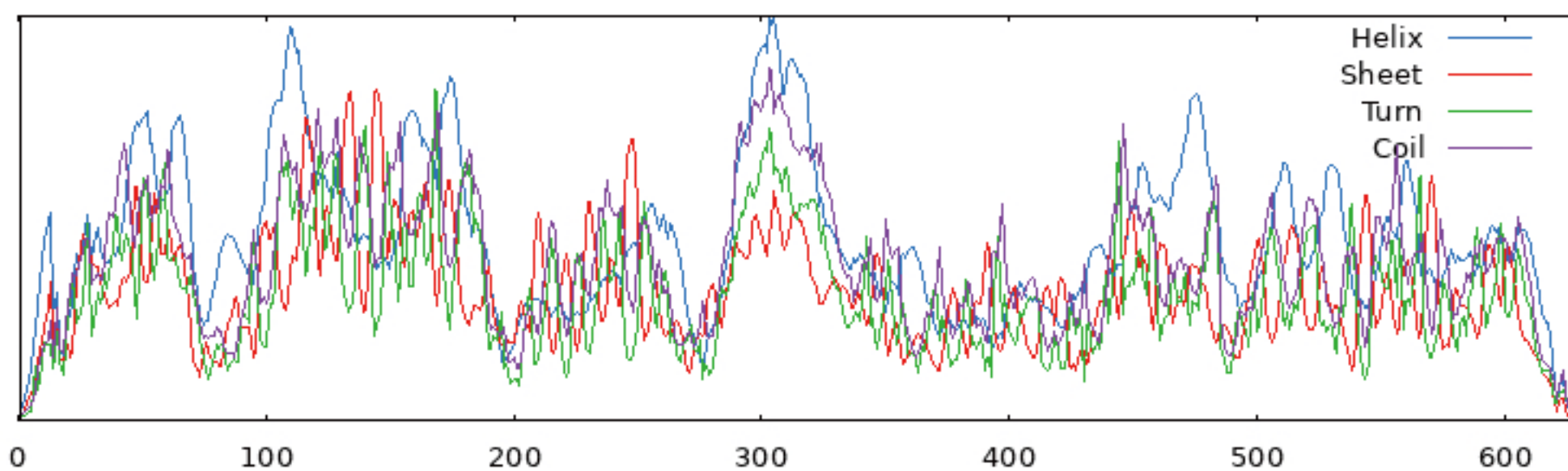

Supplement: Supplementary file 1 [file ijms-26-09908-s001.zip › Figure.s3.pdf]

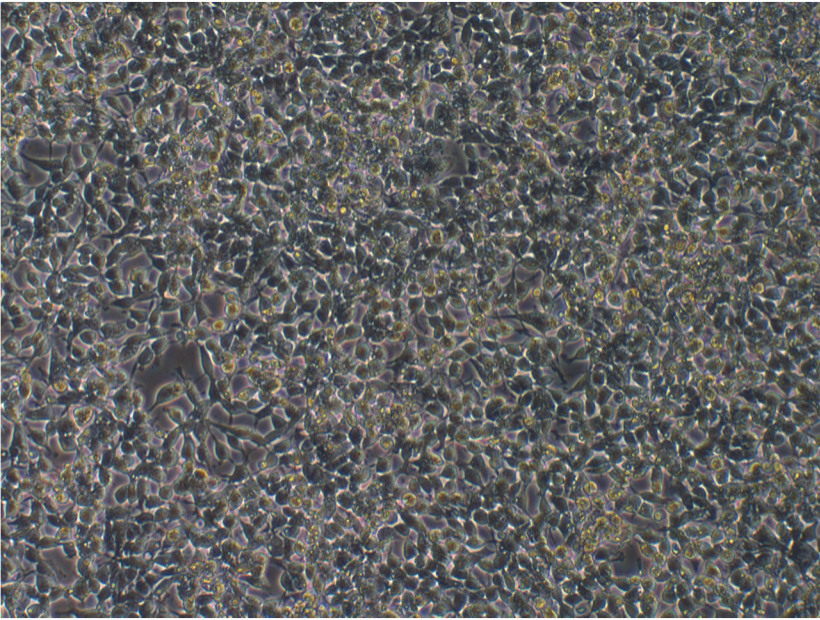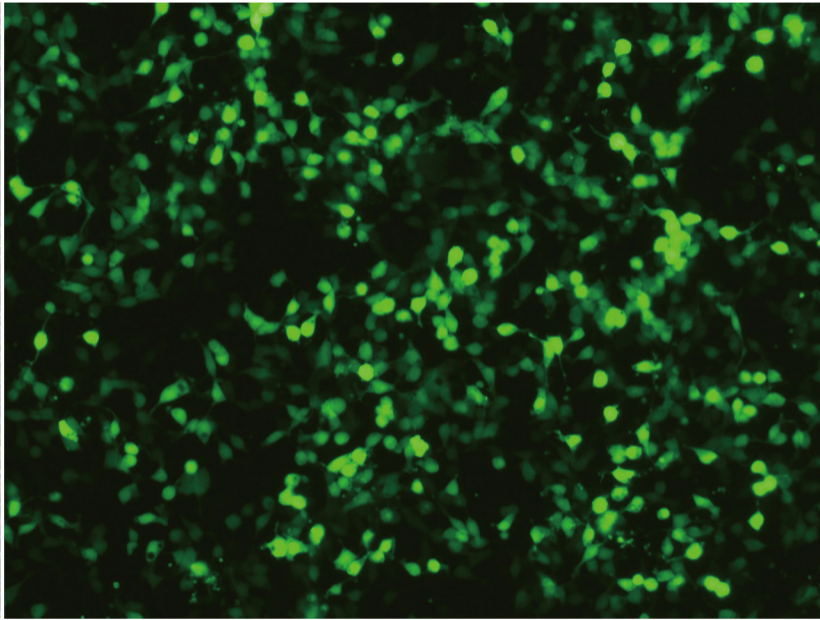

Supplement: Supplementary file 1 [file ijms-26-09908-s001.zip › Figure.s4.pdf]
