# Supplementary material for: Investigation of the Role of miR-1236-3p in Heat Tolerance of American Shad (Alosa sapidissima) by Targeted Regulation of hsp90b1
Source: Int J Mol Sci. 2025 Oct 11;26(20):9908. doi: 10.3390/ijms26209908 (PMC12564195; doi:10.3390/ijms26209908)
Supplement: Supplementary file 1 [file Figure.s2.pdf › Figure.s2.pdf]

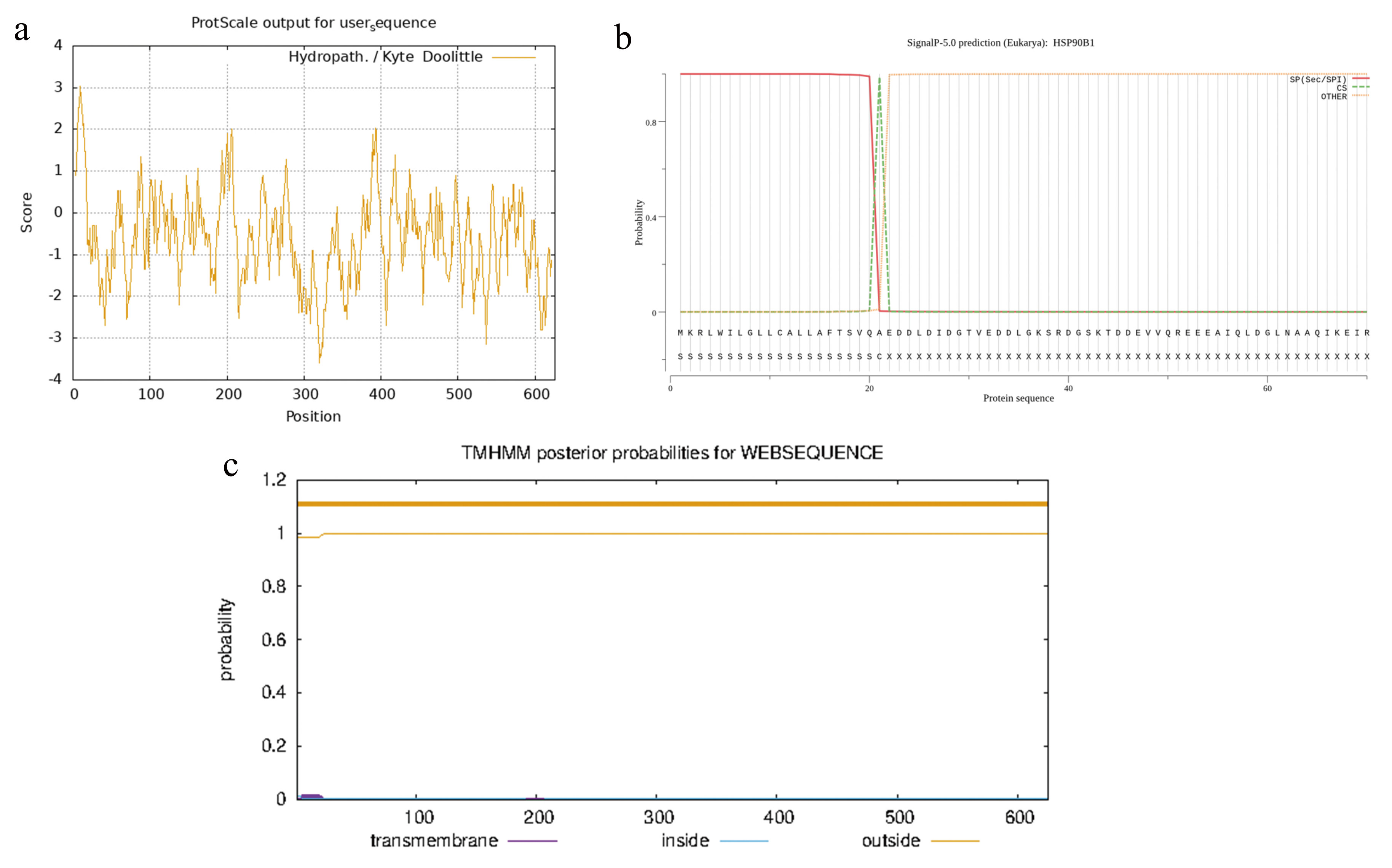
a
ProtScale output for user sequence
Hydropath. / Kyte Doolittle
Score
Position
b
SignalP-5.0 prediction (Eukarya): HSP90B1
SP(Sec/SPI)
CS
OTHER
Probability
M K R L W I L G L L C A L L A F T S V Q A E D D L D I D G T V E D D L G K S R D G S K T D D E V V Q R E E E A I Q L D G L N A A Q I K E I R
S S S S S S S S S S S S S S S S S S S S C X X X X X X X X X X X X X X X X X X X X X X X X X X X X X X X X X X X X X X X X X X X X X X X X X X X
Protein sequence
c
TMHMM posterior probabilities for WEBSEQUENCE
probability
transmembrane
inside
outside
